# Annealing Gold Foil

**Published:** 1848-01

**Authors:** 


### ANNEALING GOLD FOIL.

Sometimes it happens, that the Gold Foil which we buy, and especially the low numbers, is not well annealed, producing obstacles to success in the hands of the best operators. The defect may be remedied by the following simple contrivance: place a sheet of foil between two pieces of fine wire gauze, larger (for convenience) than the foil, and heat it over the flame of alcohol, ignited in a saucer. The flame will not penetrate the gauze, but renders the wire red hot, while the caloric of the wire is given off to the foil, until it is annealed, without any danger of fusion.—St. L. Ed.

